# What factors shape genetic diversity in cetaceans?

**DOI:** 10.1002/ece3.3727

**Published:** 2018-01-03

**Authors:** Felicia Vachon, Hal Whitehead, Timothy R. Frasier

**Affiliations:** ^1^ Department of Biology Dalhousie University Halifax NS Canada; ^2^ Department of Biology and Forensic Sciences Programme Saint Mary's University Halifax NS Canada

**Keywords:** cetacea, encephalization quotient, genetic diversity, matrilineal social systems, population size

## Abstract

Understanding what factors drive patterns of genetic diversity is a central aspect of many biological questions, ranging from the inference of historical demography to assessing the evolutionary potential of a species. However, as a larger number of datasets have become available, it is becoming clear that the relationship between the characteristics of a species and its genetic diversity is more complex than previously assumed. This may be particularly true for cetaceans, due to their relatively long lifespans, long generation times, complex social structures, and extensive ranges. In this study, we used microsatellite and mitochondrial DNA data from a systematic literature review to produce estimates of diversity for both markers across 42 cetacean species. Factors relating to demography, distribution, classification, biology, and behavior were then tested using phylogenetic methods and linear models to assess their relative influence on the genetic diversity of both marker types. The results show that while relative nuclear diversity is correlated with population size, mitochondrial diversity is not. This is particularly relevant given the widespread use of mitochondrial DNA to infer historical demography. Instead, mitochondrial diversity was mostly influenced by the range and social structure of the species. In addition to population size, habitat type (neritic vs. oceanic) had a significant correlation with relative nuclear diversity. Combined, these results show that many often‐unconsidered factors are likely influencing patterns of genetic diversity in cetaceans, with implications regarding how to interpret, and what can be inferred from, existing patterns of diversity.

## 1 INTRODUCTION

From its beginnings, the primary goal of population genetics has been to understand what factors shape patterns of genetic diversity within and among populations. Prior to the first studies quantifying allozyme variation in wild populations, there was debate about the magnitude of genetic diversity expected (e.g., Avise, 1994; Dobzhansky, 1955). Those with the “classical view” thought that little variation would exist within populations because selection would drive beneficial alleles to fixation and remove those that were detrimental. Those with the “balance view,” on the other hand, thought that populations would have an abundance of variation through balancing selective forces such as heterozygote advantage. The finding and characterization of substantial intrapopulation variation by the first electrophoretic studies (Harris, 1966; Lewontin & Hubby, 1966), and continuing thereafter, have dramatically changed these views, and have lead to the idea that many alleles segregate within populations as if they were neutral or nearly neutral (Kimura, 1968a,b; Ohta, 1973). Under this model, the key factors shaping patterns of genetic diversity include the underlying mutation rate, migration rates among populations, and population size (e.g., Griffiths & Tavare, 1994; Kimura & Ohta 1971; Kingman, 1982).

Analytical methods in population genetics, and their application, have flourished under the assumptions of the neutral model. Specifically, based on the idea that population size and genetic variability are closely associated, numerous methods have been developed to infer population size (contemporary and/or historical) based on existing patterns of diversity (e.g., Beaumont, 1999; Beerli & Felsenstein, 1999; Wu & Drummond, 2011). These methods have been influential in a wide range of fields. For example, inferences of historical population sizes based on DNA sequences suggest that the pre‐exploitation sizes of several whale populations were orders of magnitude larger than had been estimated based on whaling records, with subsequent impacts on our understanding of the carrying capacity of historical environments and the recovery rate and potential of the affected species (Alter, Rynes, & Palumbi, 2007; Roman & Palumbi, 2003). Similarly, such approaches have been key in teasing apart the relative role of climatic changes and human hunting on the extinction of Pleistocene megafauna (Shapiro et al., 2004; Stiller et al. 2010). The tide is turning again, however, as an increasing number of studies show that levels of genetic diversity may not be as closely associated with population size as once thought (e.g., Bazin, Glémin, & Galtier, 2006; Corbett‐Detig, Hartl, & Sackton, 2015). While first pointed out by Richard Lewontin in 1974 (Lewontin, 1974), this idea and its implications are gaining more traction now, given the dependency of many modern analytical methods on the assumption of a close relationship between genetic diversity and population size, and the subsequent implications on the interpretation of results from studies applying these methods.

Cetaceans (whales, dolphins, and porpoises) represent a group of mammals for which the association between genetic diversity and population size may be particularly weak. For example, their long lifespans and generation times mean that populations may never reach mutation–drift equilibrium in between major changes in distribution, population structure, and abundance. The bowhead whale (*Balaena mysticetus*), for example, lives for over 200 years (George & Bocktoce, 2008; George et al., 1999) and has a generation time of approximately 37 years (Taylor, Martinez, Gerrodette, Barlow, & Hrovat, 2007). Varvio, Chakraborty, and Nei (1986) showed that under the neutral model, even with no migration, it takes ~4*N*_*e*_ generations for intrapopulation heterozygosity to reach equilibrium once a population has been split into multiple populations, and it takes slightly longer for the associated *F*_ST_ values to reach equilibrium. Thus, for a bowhead whale population with an effective population size (*N*_*e*_) of just 1,000 individuals, it would take ~148,000 years to reach equilibrium. Given that major environmental changes, such as ice ages, occur in cycles much shorter than this, it is unlikely that the genetic characteristics in any bowhead whale population are *ever* representative of equilibrium conditions. Although the bowhead whale is an extreme example, the same is true for many cetaceans. Most species examined by Taylor et al. (2007) have generation times of 10 years or longer. Again, taking a conservative estimate of 1,000 individuals for *N*_*e*_ results in an expectation of ~40,000 years to reach equilibrium, which again is a longer time frame than that in which major ecological shifts tend to occur. Thus, rather than being reliable indicators of current conditions, the contemporary patterns of genetic diversity in cetacean species are likely a mishmash of the slow accumulation of signatures from current conditions, as well as extensive residual signatures of multiple (and perhaps conflicting) events in the past.

A number of factors other than population size are also known to influence patterns of genetic diversity, the effects of which may also be amplified in cetaceans. For example, the social structure, and related movement and reproductive patterns of a species can have far‐reaching impacts on diversity, with different effects on the nuclear and mitochondrial genomes (e.g., Chesser, 1991; Chesser & Baker, 1996; Hoelzel, 1998). Many cetacean species have complex social structures that likely have a large influence on patterns of genetic diversity. Indeed, some species—such as killer whales (*Orcinus orca*) and sperm whales (*Physeter macrocephalus*)—have matrilineal social systems and reduced levels of mitochondrial diversity (Whitehead, Vachon, & Frasier, 2017). There are several mechanisms by which matrilineality could have led to reduced mitochondrial genetic diversity. These include cultural hitchhiking, by which diversity at a neutral genetic locus is reduced due to selection on culturally inherited traits that are being transmitted in parallel (Whitehead, 1998), as well as bottlenecks or selection in culturally specialized killer whale ecotypes founded by matrilines (Foote et al., 2016). Mechanisms such as cultural hitchhiking and ecotype‐specific gene selection are not mutually exclusive and could interact in shaping genetic diversity in species with complex social and population structures (Whitehead et al., 2017).

Given that cetaceans represent some of the extremes in the animal kingdom, with respect to lifespans, generation times, movement abilities and ranges, and social complexity, they represent scenarios where the factors influencing patterns of genetic diversity may differ substantially from those commonly assumed, and taken into consideration, when interpreting and making inferences from genetic characteristics. To address this issue, and to gain more insight into what factors are shaping patterns of genetic diversity in cetaceans, we analyzed data synthesized from a systematic literature review. Specifically, we searched the literature for studies publishing estimates of mitochondrial and/or microsatellite diversity for all populations and species of marine cetaceans. We related these diversity estimates to 10 classes of factor that could potentially influence diversity: (1) population size; (2) IUCN status and trend; (3) exploitation history; (4) phylogeny; (5) latitudinal range; (6) habitat type (neritic and oceanic); (7) body size (maximum length); (8) generation time and lifespan; (9) brain size; and (10) social structure. The rationale behind the choice of these classes of factor is explained below:

1. *Population size*. Species with larger population size are expected to have higher genetic diversity due to reduced genetic drift and inbreeding depression (Leffler et al., 2012; Wright, 1931). This effect has been widely documented (Frankham et al. 2002; McCusker & Bentzen, 2010).
2. *IUCN status and trend*. High genetic diversity is expected in healthy populations as it is assumed to correlate with resilience (Amos & Harwood, 1998), while low genetic diversity is expected in species with small or declining populations because of drift or inbreeding depression (Leffler et al., 2012). IUCN status and Population trend indicate the conservation status of a species; endangered species and species with declining populations are expected to have lower genetic diversity than those of least concern (Spielman, Brook, Frankham, & Schaal, 2004).
3. *Exploitation history*. Whaling caused dramatic population declines that could have led to genetic bottlenecks (Amos & Harwood, 1998). We expect lower genetic diversity in harvested species (Jackson et al., 2014).
4. *Phylogeny*. Differentiation in the mutation rate among different lineages has been suggested as a cause of differences between genetic diversity in mammalian orders (Nabholz, Mauffrey, Bazin, Galtier, & Glemin, 2008).
5. *Latitudinal range*. Latitudinal range is a measure of the diversity of habitats within a species’ range. With greater habitat diversity, we would expect greater genetic diversity due to drift in isolated habitats, selection and a larger gene pool. Hence, species with larger ranges are expected to have more genetic diversity (DeWoody & Avise, 2000; Doyle, Hacking, Willoughby, Sundaram, & Dewoody, 2015; Leffler et al., 2012).
6. *Habitat type*. Habitat has been shown to impact genetic diversity in terrestrial mammals and amphibians through a latitudinal gradient (Miraldo et al., 2016). Genetic diversity has also been documented to differ for freshwater and saltwater fishes (DeWoody & Avise, 2000) and for coastal and offshore populations of cetaceans (Natoli, Peddemors, & Hoelzel, 2004).
7. *Body size*. Species with smaller body size are generally more genetically diverse than larger species (Mitton & Lewis, 1989; Romiguier et al., 2014).
8. Generation time and lifespan. Longer generation time and lifespans have been suggested to correlate with lower genetic diversity (Mitton & Lewis, 1989; Romiguier et al., 2014) as genes are less frequently mixed via reproduction.
9. *Brain size*. The impact of intelligence on genetic diversity is not well documented. Wilson (1985) suggested that intelligent species diversify faster due to their ability to innovate, as well as their behavioral plasticity. However, intelligence, in its role of promoting phenotypic plasticity, could buffer species against environmental variability, reducing genetic selection.
10. *Social structure*. There is an increasing body of literature suggesting that social structure can impact cetacean's genetic diversity (Foote et al., 2016; Whitehead et al., 2017)

From these 10 classes of factor, we derived 21 factors (Table 1), which were correlated with the genetic diversity of 42 cetacean species. The only exception is *Brain size*, which was restricted to Odontocetes (toothed whales) as Mysticetes (baleen whales) have disproportionate body enlargement, making EQ (our measure of brain size) inappropriate (Marino, 2008). Generalized linear models were then used to assess the relative impact of each factor on mitochondrial and nuclear diversity.

**Table 1:** Factors potentially predicting cetacean genetic diversity, with number of species for which this factor could be determined (*n*). *Whaling1* differs from *Whaling2* as it is an index based on the historical whaling information presented in Perry, Demaster, and Silber (1999), while *Whaling2* is a binary variable indicating whether the species has been harvested through whaling or not. *Ocean1* is the number of oceans included in the species’ range (up to 5), and *Ocean2* indicates whether the species is found exclusively in the Atlantic, Pacific, or in both

Factor	Type	Levels/notes	Restrictions	*n*	Reference
Current population size	Quantitative	Data were logged	Population known within a factor of 5	35	IUCN Red List ver3.1 (2016)
Current IUCN status	Categorical	Least Concern (LC) Near Threatened (NT) Vulnerable (VU) Endangered (EN)	Data Deficient (DD) species not included	27	IUCN Red List ver3.1 (2016)
IUCN status from 1990s	Categorical	(Same as above)	DD species not included	26	IUCN Red List ver3.1 (2016)
Population trend	Categorical	DecreasingStableIncreasing		11	IUCN Red List ver3.1 (2016)
Whaling1	Categorical	NoneSomeExtensive		42	From Perry et al. (1999)
Whaling2	Categorical	HarvestedNot harvested		42	From Perry et al. (1999)
Mysticetes/odontocetes	Categorical			42	Society for Marine Mammalogy (2016)
Cetacean families	Categorical	BalaenidaeBalaenopteridaeDelphinidaeMonodontidaeEschrichtiidaeZiphiidaeKogiidaePhocoenidaePhyseteridaePontoporiidae		42	Society for Marine Mammalogy (2016)
Latitudinal range	Quantitative	Total number of degrees of latitude within the species’ distribution		40	Charts from Folkens, Folkens, Stewart, Clapham, and Powell (2002)
Hemisphere	Categorical	SouthernNorthern		42	Map from IUCN Red List (2016)
Ocean1	Quantitative	Number of oceans overlapping the species’ range (1–5)		42	Map from IUCN Red List (2016)
Ocean2	Categorical	AtlanticPacificBoth		42	Map from IUCN Red List (2016)
Habitat	Categorical	NeriticOceanicBoth		42	IUCN Red List (2016)
Temperature	Categorical	Tropical/temperatePolarCosmopolitan		42	Folkens et al. (2002)
Maximum length	Quantitative	m	Female	38	Folkens et al. (2002)
Generation time	Quantitative	yr		37	Folkens et al. (2002)
Lifespan	Quantitative	yr		31	Folkens et al. (2002)
Encephalization Quotient	Quantitative	EQ_0.67_ by Jerison (1973)	Only Odontocetes	22	Marino (2008); Marino et al. (2004)
Group size	Categorical	1: solitary or pairs2: 3–10 individuals3: 10–50 individuals4: hundreds		42	Folkens et al. (2002)
Breeding strategy	Categorical	CongregateDisperse	Only Mysticetes	11	
Social structure	Categorical	MatrilinealNot matrilineal	Matrilineal if female offspring stays with mother for entire lifetime	42	Whitehead et al. (2017)

## 2 MATERIALS AND METHODS

### 2.1 Genetic diversities of cetacean species

Genetic diversity data for nonriverine cetacean species (as listed by the Society for Marine Mammalogy [Committee on Taxonomy 2016]) were derived as described below. These methods are the same as in Whitehead et al. (2017).

Nucleotide diversities in the control region of the mtDNA (π in %) were obtained from Table 1 of Alexander et al. (2013) and a systematic literature review using Web of Science^™^ (search terms “TS=(mitochondrial OR mtDNA) AND TS=([common name] OR [Latin name])”). Microsatellite data were obtained from the Supplementary Material of Bourret, Mace, Bonhomme, and Crouau‐Roy (2008) for papers published between 1989 and 2007, and a systematic literature review on Web of Science^™^ using the terms “TS=(microsatellite*) AND TS=(whale* OR dolphin* OR porpoise* or cetacean*)” that covered the years 2008–2015. For each published use of each microsatellite on each cetacean species, we tabulated the microsatellite name, the species name, the number of individuals tested, the number of alleles found, and whether the microsatellite was ascertained on that specific species. For both the mitochondrial and microsatellite data, the datasets were also further divided into Rangewide (“O”) and Regional (“R”) samples. Data were considered as Rangewide if the samples covered 25% or more of the species range or an entire ocean basin, and Regional otherwise. The rationale for calculating diversity in these two different ways is to account for population structure. Briefly, if population structure is having a large impact on genetic diversity within a species, then the best‐fit model for the regional dataset should differ from that for the rangewide dataset. Moreover, comparing the models and estimated effects between the regional and rangewide datasets provides useful information on factors influencing diversity at smaller versus larger scales.

Rangewide and Regional estimates of mtDNA diversity for each species were calculated as the means of all published estimates of π with sample size greater than or equal to 100 (as in Alexander et al., 2013).

Rangewide and Regional estimates of microsatellite diversity for each species were calculated as in Whitehead et al. (2017) using a methodology that accounted for differences in allelic richness between microsatellite loci, sample size, and ascertainment bias. First, the number of alleles recorded for a particular species at a particular locus in a particular study was corrected for sample size using:

$$\begin{array}{cc} & \text{Corrected no.alleles}\;=\\ & \frac{\text{No.alleles for this study, species and microsatellite}\;*\;(1\;+\;\text{sample size}\;*\;\mu )}{\text{sample size}\;*\;\mu } \end{array}$$

In this, μ was estimated by fitting a simple asymptotic model, giving μ = 0.1975 for the Regional (micR) data and μ = 0.2447 for the Rangewide (micO) data (Whitehead et al., 2017). Then, a linear mixed‐effects model was fitted to all the data:

$$\begin{array}{cc} & \text{Log (Corrected no. of alleles)}∼\text{Species effect + Microsatellite effect}+\\ & \;\text{Ascertainment effect} \end{array}$$

Here, the Species effect is a fixed effect for each species, the Microsatellite effect is a random effect over microsatellite loci, and the Ascertainment effect is a binary fixed effect (1 if the microsatellite was ascertained on the species, 0 if not). We only included microsatellite loci that had been used on at least five different species, and species with analyses using at least four microsatellites, each with at least two alleles and a sample size greater than five individuals. The species effect in the linear mixed‐effects model, our estimate of relative nuclear genetic diversity, corresponds to the log of the actual divided by expected allelic diversity for each species relative to other cetaceans, controlling for sample size, the diversities of the different microsatellites, and ascertainment bias. Thus, a value below zero indicates lower than expected relative nuclear genetic diversity compared to other cetaceans and above zero greater than expected.

These procedures resulted in relative genetic diversity estimates for: 30 cetacean species for the Regional microsatellite dataset (micR), 22 species for the Rangewide microsatellite dataset (micO), 23 species for the Regional mitochondrial dataset (πR) and 27 species for the Rangewide mitochondrial dataset (πO). In total, 42 different cetacean species were considered in the analysis: 31 odontocetes (toothed whales) and 11 mysticetes (baleen whales). The genetic diversity data are tabulated in Table S1.

### 2.2 Predictive factors

Twenty‐one different predictive factors were considered in this study (Table 1). Procedural information for these factors, as well as values for each species, are given in the (Table S1 and Appendix S1).

### 2.3 Statistical analysis

Boxplots or beanplots (Kampstra, 2008) for each categorical variable were created for all four measures of genetic diversity (πR, πO, micR, micO). One‐way ANOVAs were then used to test the null hypothesis that genetic diversity does not differ between levels of the factor for each genetic marker at both the Regional and Rangewide scales. In order to measure effect size, Cohen's *d* was calculated for factors with two levels (Cohen, 1992) and ω^2^ for factors with more than two levels (Hays, 1963). ω^2^ was chosen over η^2^ because it is a less biased measure when the sample size is small (Carroll & Nordholm, 1975; Keselman, 1975). The effect was considered strong if *d *>* *0.8 or ω^2^>0.14 (Cohen, 1992). Measures of genetic diversity were plotted against continuous factors in scatterplots, and *r* was calculated as a measure of effect size. Correlations with |*r*| > .4 were considered strong. To account for phylogenetic correlation, the effects of continuous factors were also tested using phylogenetic independent contrasts (PICS). PICS removes the phylogenetic bias by correlating the independent contrasts (differences in value at each node of the phylogenetic tree) instead of only correlating the values presented at the “end” of the phylogenetic tree (Felsenstein, 1985). The phylogeny used for this analysis is from Steeman et al. (2009).

In order to assess the relative importance of each factor on cetacean genetic diversity, we created general linear models (GLMs) for each Regional and Rangewide genetic diversity marker (πR, πO, micR, micO). Backwards/and forwards stepwise regression using Akaike Information Criterion (AIC) was used to discriminate between the different models. AIC is a measure of the Kullback–Leibler Information, which is the distance between two models: in this case, the model being tested and reality. Therefore, when comparing between models, the most efficient model at predicting reality is the one with the lowest AIC (Akaike, 1974). *EQ* and *Population trend* were not included in these models because they reduced the species’ sample size too dramatically. Simpler models were favored over more complex ones if their difference in AIC were less than ~5.0. Phylogenetic generalized least squares (PGLS) was used to obtain the respective *F*‐values and *p*‐values of the factors included in our GLMs as it accounts for the nonindependence of both continuous and categorical phylogenetic values in linear models (Grafen, 1989). (This is not possible with PICS, as PICS is restricted to univariate continuous data [Felsenstein, 1985].) An Ornstein–Uhlenbeck (OU) process (Hansen, 1997) was preferred over Brownian motion since it incorporates natural selection and drift into the model, accounts for selective optimums, and is considered a more accurate process (Butler & King, 2004). We computed Pagel's λ for the PGLS models in order to quantify the maximum likelihood of phylogenetic autocorrelation (Pagel, 1999). If λ = 0, the values are considered independent from phylogeny and the further from 0, the more important the phylogeny is (Münkemüller et al., 2012). We choose Pagel's λ over Abouheif's *C*_mean_, Moran's *I* and Blomberg's *K* because it is considered a better alternative, allowing for more complex modes of evolution and resulting in less type 1 error (Münkemüller et al., 2012).

## 3 Results

### 3.1 Factors tested by themselves

When tested on their own, most of the 21 factors considered in this study were not significantly related to measures of genetic diversity (at α = 0.05) and did not show strong effect sizes (Tables 2 and 3). Moreover, none were significantly related to measures of genetic diversity for both the nuclear and mitochondrial markers (Tables 2 and 3).

**Table 2:** Results of the phylogenetic independent contrasts analyses evaluating the impact of continuous factors on cetacean genetic diversity. *n* corresponds to the species sample size for each combination of factor and genetic marker, πR to Regional mitochondrial control region nucleotide diversity, πO to Rangewide mitochondrial control region nucleotide diversity, micR to Regional microsatellite genetic diversity estimates and micO to Rangewide microsatellite genetic diversity estimates

Factor	Marker	*n*	*F*‐value	*p*‐value	Correlation coefficient (*r*)
Population size	πR	20	0.96	.341	0.224
	πO	23	0.04	.839	0.045
	micR	27	**6.25**	**.020**	**0.425**
	micO	21	**18.60**	**.000**	**0.703**
Population size without matrilineal species	πR	16	1.36	.265	0.297
	πO	18	0.05	.826	0.056
Latitudinal range	πR	23	0.31	.586	0.120
	πO	27	0.18	.679	0.083
	micR	28	**5.53**	**.026**	**0.419**
	micO	22	0.17	.684	0.092
Latitudinal range without matrilineal species	πR	19	3.84	.066	*0.429*
	πO	22	1.55	.228	0.268
Maximum length	πR	22	0.13	.720	0.088
	πO	27	0.46	.506	0.134
	micR	28	0.00	.969	0.008
	micO	21	0.01	.906	0.027
Generation time	πR	22	2.32	.143	0.323
	πO	24	1.13	.300	0.300
	micR	26	0.42	.520	0.132
	micO	21	0.13	.719	0.083
Lifespan	πR	19	1.86	.190	0.314
	πO	22	0.31	.581	0.125
	micR	24	0.53	.475	0.153
	micO	19	0.10	.760	0.075
Encephalization Quotient	πR	12	3.69	.084	*0.519*
	πO	11	*5.48*	*.044*	*0.615*
	micR	12	1.50	.248	0.361
	micO	10	1.25	.296	0.367

Significant results or those with strong effect sizes are indicated in bold for relative nuclear genetic diversity data and italics for mitochondrial genetic diversity data.

**Table 3:** Results of the ANOVAs evaluating the impact of categorical factors on cetacean genetic diversity with their corresponding Cohen's *d* (two‐level factor) or η^2^ (more than two levels). *n* corresponds to the species sample size for each combination of factor and genetic marker, and descriptions of πR, πO, micR and micO can be found in the legend of Table 2

Factor	Marker	*n*	*F*‐value	*p*‐value	Effect size	
					Cohen's *d*	ω^2^
Current IUCN status	πR	14	0.70	.601		0.069
	πO	16	1.03	.415		0.024
	micR	22	1.48	.234		**0.196**
	micO	18	2.10	.126		**0.151**
IUCN status from 1990s	πR	16	0.83	.560		*0.222*
	πO	20	0.07	.999		*0.227*
	micR	21	**2.99**	**.026**		**0.153**
	micO	18	1.83	.163		**0.233**
Population trend	πR	7	0.85	.492		0.044
	πO	8	0.83	.516		*0.145*
	micR	7	**30.30**	**.004**		**0.893**
	micO	7	**8.86**	**.034**		**0.692**
Whaling1	πR	23	0.39	.539		0.027
	πO	27	0.16	.695		0.032
	micR	30	0.44	.515		0.019
	micO	22	1.59	.222		0.026
Whaling2	πR	23	0.98	.333	0.416	
	πO	27	0.00	.987	0.006	
	micR	30	0.90	.352	0.359	
	micO	22	2.12	.161	0.623	
Mysticetes against Odontocetes	πR	23	1.52	.231	0.586	
	πO	27	3.26	.083	0.719	
	micR	30	3.36	.077	0.757	
	micO	22	0.11	.741	0.153	
Cetacean families	πR	23	0.87	.551		0.041
	πO	27	1.45	.241		0.119
	micR	30	1.11	.386		0.021
	micO	22	0.19	.983		**0.347**
Hemisphere	πR	23	1.84	.185		0.068
	πO	27	0.85	.441		0.011
	micR	30	1.30	.288		0.020
	micO	22	**3.90**	**.038**		**0.209**
Ocean1	πR	23	0.58	.455		0.019
	πO	27	1.59	.220		0.021
	micR	30	**9.68**	**.004**		**0.224**
	micO	22	0.75	.396		0.011
Ocean2	πR	23	0.38	.689		0.057
	πO	27	0.89	.422		0.008
	micR	30	**11.80**	**.000**		**0.419**
	micO	22	2.14	.146		0.094
Habitat	πR	23	2.03	.158		0.082
	πO	27	0.24	.787		0.059
	micR	30	**4.19**	**.026**		**0.175**
	micO	22	**3.47**	**.052**		**0.183**
Temperature	πR	23	1.77	.196		0.063
	πO	27	0.57	.571		0.033
	micR	30	1.07	.356		0.004
	micO	22	2.21	.137		0.099
Group size	πR	23	0.66	.426		0.015
	πO	27	2.67	.115		0.058
	micR	30	0.01	.930		0.034
	micO	22	2.72	.115		0.072
Breeding strategy	πR	6	0.03	.868	0.144	
	πO	10	1.07	.331	0.655	
	micR	8	0.24	.644	0.355	
	micO	7	0.28	.621	0.402	
Social structure	πR	23	*9.95*	*.005*	*1.730*	
	πO	27	*5.83*	*.023*	*1.200*	
	micR	30	0.15	.699	0.210	
	micO	22	1.35	.258	0.723	

Significant results or those with strong effect sizes are indicated in bold for relative nuclear genetic diversity data and italics for mitochondrial genetic diversity data.

**Figure 1:**
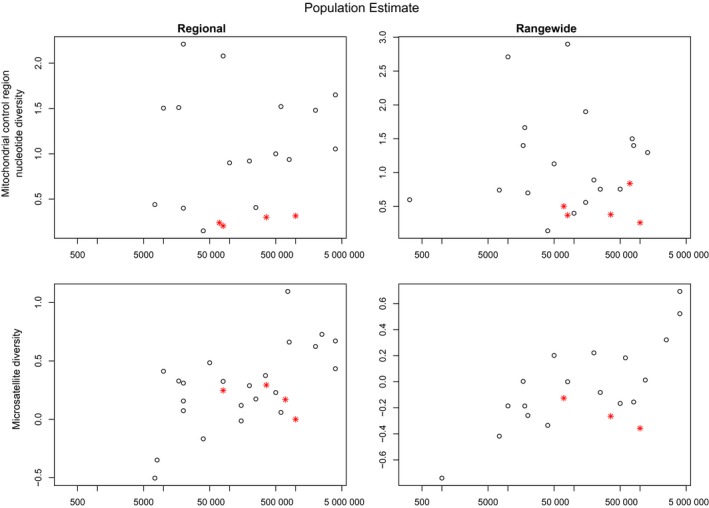
Mitochondrial and relative nuclear genetic diversity of cetacean species plotted against approximate population size for both “Regional” and “Rangewide” datasets. Matrilineal species are designated by a red star

**Figure 2:**
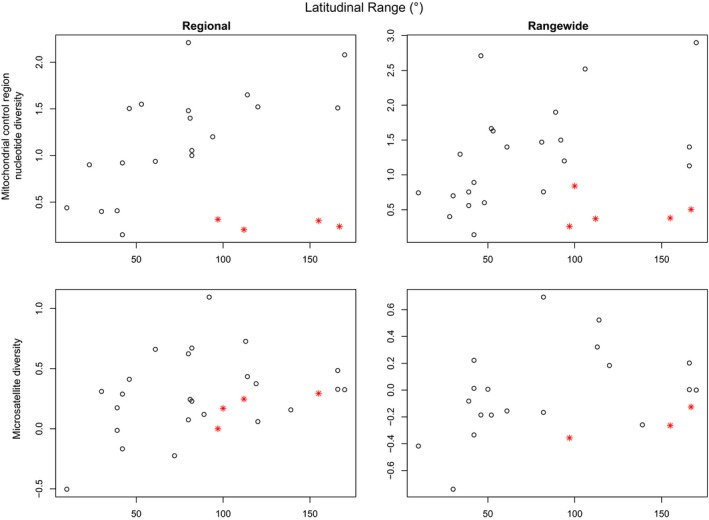
Mitochondrial and relative nuclear genetic diversity of cetacean species plotted against latitudinal range (in degrees) for both “Regional” and “Rangewide” datasets. Matrilineal species are designated by a red star

**Figure 3:**
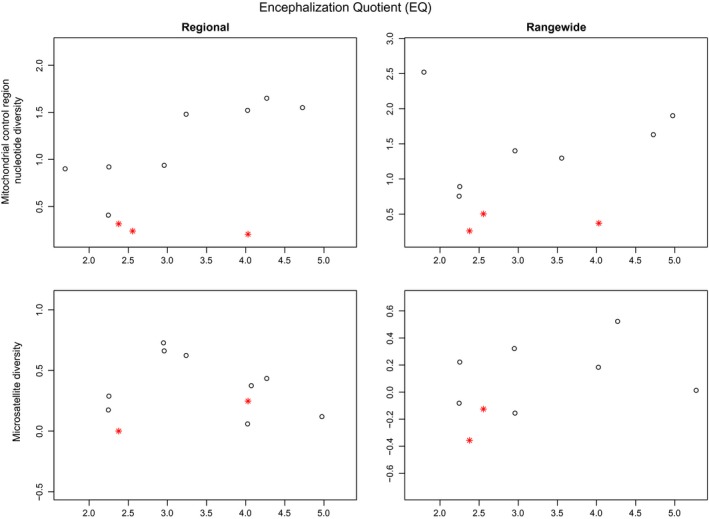
Mitochondrial and relative nuclear genetic diversity of cetacean species plotted against encephalization quotient (EQ_0.67_) for both “Regional” and “Rangewide” datasets. Matrilineal species are designated by a red star

Three of the continuous variables showed significant correlation with at least one of the genetic markers (Table 2). According to the PICS analysis, *Population size* was only correlated with nuclear DNA genetic diversity. The correlation was positive for both Regional and Rangewide nuclear data. As expected, more abundant populations tend to have greater relative nuclear genetic diversity (Figure 1). *Latitudinal range* was only significantly correlated with the Regional microsatellite data. However, when matrilineal cetaceans were removed from the PICS analysis, the correlation coefficient dramatically increased for the mitochondrial datasets: going from 0.120 to 0.429 (πR) and 0.0835 to 0.268 (πO) (Table 2, Figure 2). This result further highlights the importance of social structure in determining mitochondrial genetic diversity. *EQ*, while having relatively high correlation coefficients with the diversity of all genetic markers (0.361 for micR, 0.367 for micO, 0.519 for πR, and 0.615 for πO) was only significant for πO (Table 2, Figure 3).

**Figure 4:**
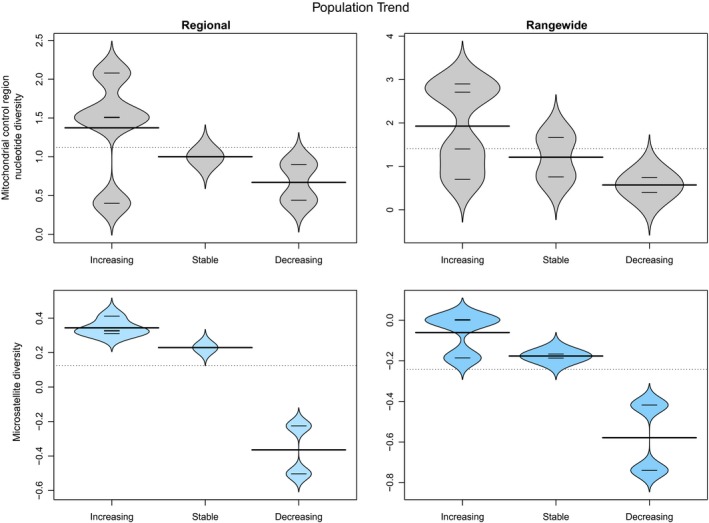
Beanplot of mitochondrial and relative nuclear genetic diversity of cetacean species according to their IUCN Population trend (Increasing, Stable or Decreasing) for both “Regional” and “Rangewide” datasets. Significant results (α = 0.05) are colored in blue

**Figure 5:**
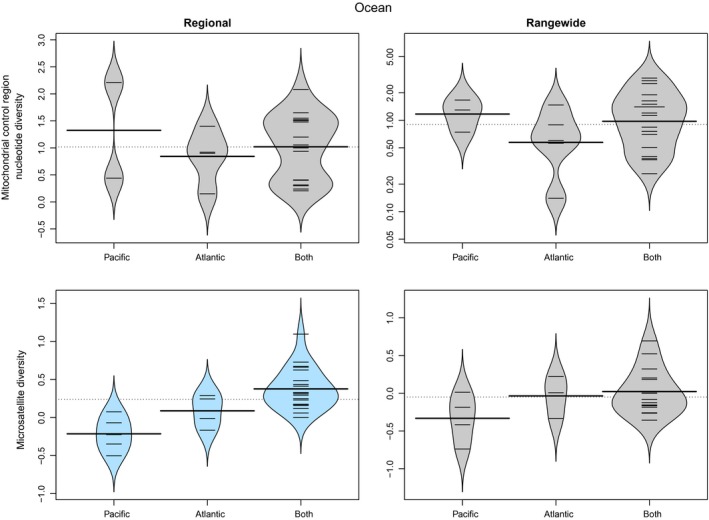
Beanplot of mitochondrial and relative nuclear genetic diversity of cetacean species according to their distribution in either or both of the Pacific and Atlantic Oceans for both “Regional” and “Rangewide” datasets. Significant results (α = 0.05) are colored in blue

**Figure 6:**
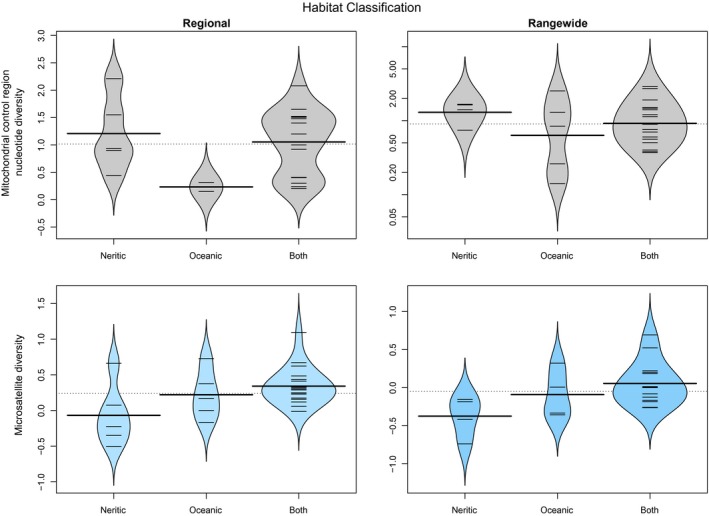
Beanplot of mitochondrial and relative nuclear genetic diversity of cetacean species according to the classification of their habitat for both “Regional” and “Rangewide” datasets. Neritic habitat corresponds to habitat within the continental shelf while oceanic habitat is defined as beyond it. Significant results (α = 0.05) are colored in blue

**Figure 7:**
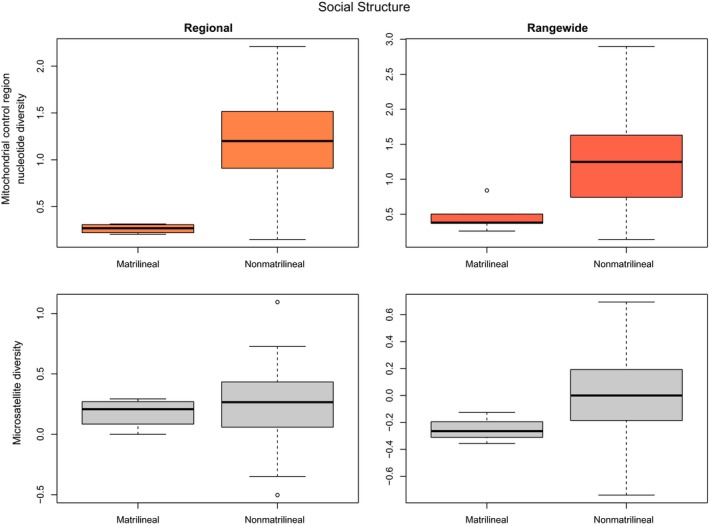
Boxplots of mitochondrial and relative nuclear genetic diversity of cetacean species according to their social structure—whether they possess a matrilineal social system or not, for both “Regional” and “Rangewide” datasets. Significant results (α = 0.05) are colored in orange

Seven of the categorical factors from three classes had a significant (ANOVA: α < 0.05) and strong effect on one or more measures of cetacean genetic diversity (Table 3): *IUCN status from 1990s, Population trend*,* Hemisphere*,* Ocean*,* Ocean2*,* Habitat* and *Social structure*. *IUCN status from 1990s* and *Population trend* as reported by IUCN are related. The more endangered species, with decreasing populations, had generally lower genetic diversity (e.g., Figure 4), although sample sizes were small and this was only significant for the nuclear markers (Table 3). Rangewide relative nuclear diversity was higher in the Northern Hemisphere species compared to those in the Southern Hemisphere, while regional relative nuclear diversity was higher for Atlantic species as compared with Pacific species; but as might be expected, highest of all for species in both hemispheres and both major oceans (Figure 5). Species that inhabit neritic habitats (within the continental shelf) had lower relative nuclear genetic diversity than species inhabiting the open ocean. Species with distributions overlapping both neritic and oceanic habitats had the highest genetic diversity (Figure 6). The only categorical variable that had a significant correlation with mitochondrial genetic diversity was *Social structure*. This factor was highly significant for both Regional and Rangewide datasets (Table 3). In both cases, matrilineal species had significantly lower mitochondrial genetic diversity than nonmatrilineal species (Figure 7).

### 3.2 Models

The best (using either AIC or, accounting for phylogenetic relationships, PGLS) model for both Regional and Rangewide mitochondrial genetic diversity data included the two factors *Social structure* and *Latitudinal range* (Tables 4 and 5). Diversity was higher for species with greater latitudinal range and lower for matrilineal ones (Figures 2 and 7).

**Table 4:** Selected general linear model results and their associated sample size in number of species (*n*), and AIC. “1” corresponds to a null model and descriptions of πR, πO, micR, and micO can be found in the legend of Table 2

Genetic marker	Model	*n*	AIC
πR	1	23	−20.88
	Social structure		−27.80
	Social structure + Latitudinal range		−38.58
πO	1	27	−15.19
	Social structure		−18.85
	Social structure + Latitudinal range		−23.42
micR	1	27	−58.18
	Ocean2		−69.21
	Ocean2 + Population size		−71.39
	Ocean1 + Population size		−70.82
micO	1	21	−45.73
	Population size		−60.07
	Population size + Habitat		−66.44

**Table 5:** Phylogenetic generalized least squares analysis results and their associated sample size in number of species (*n*) and Pagel's λ value. Descriptions of πR, πO, micR, and micO can be found in the legend of Table 2

Model	*n*	Pagel's λ		*F*‐value	*p*‐value
πR ~ Social structure + Latitudinal range	23	0.20	Intercept	117	<.0001
			Social structure	14.5	.0011
			Latitudinal range	13.3	.0016
πO ~ Social structure + Latitudinal range	27	−0.11	Intercept	91.8	<.0001
			Social structure	7.14	.0133
			Latitudinal range	6.61	.0167
micR ~ Ocean2 + Population size	27	0.07	Intercept	30.7	<.0001
			Ocean2	9.96	.0008
			Population size	3.74	.0656
micO ~ Population size + Habitat	21	−0.13	Intercept	1.65	.216
			Population size	32.8	<.0001
			Habitat	5.43	.0150

While both mitochondrial datasets had the same resulting model, this was not the case for the nuclear data. The Regional nuclear dataset was best fitted by a model including *Ocean2* and *Population size* (Tables 4 and 5). Diversity increased with population size (Figure 1) and was greatest for species in both major oceans, and least for those only in the Pacific (Figure 5). The best‐fitting model for Rangewide microsatellite data included *Population size* and *Habitat* (Table 4), with diversity increasing with population size (Figure 1) and being greatest for species found in both oceanic and neritic habitats and least for species only found in neritic habitats (Figure 6). Pagel's λ was computed for the four best‐fitting models and, in all cases, had values below 1 (Table 5), indicating that phylogeny does not greatly influence these results.

## 4 DISCUSSION

Of the 21 factors considered, mitochondrial diversity had the strongest association with *Social structure* and *Latitudinal range,* while relative nuclear genetic diversity had the strongest association with *Population size* and habitat (*Habitat* and *Ocean2*). The potential reasons for these relationships are discussed below, as well as other factors that showed weaker associations.

### 4.1 Population size

In the recent past, one common perspective in population genetics was that mitochondrial diversity should be closely correlated with population size and therefore that mitochondrial diversity could be used to make inferences on population size in situations where information on the latter was lacking. Analyses based on this assumption have flourished, with implications across a wide range of fields. However, our results are in agreement with an increasing number of studies indicating that, contrary to this common view, there is not a close relationship between population size and mitochondrial diversity (e.g., Bazin et al., 2006; Corbett‐Detig et al., 2015). This lack of a correlation remained true when the matrilineal species, which have remarkably low mitochondrial diversities, were removed from the analyses. This finding has major implications for how mitochondrial data are used and interpreted in studies of cetaceans. For instance, low levels of mitochondrial diversity are frequently interpreted as indications of a recent bottleneck (e.g., Alexander et al., 2013), often with implications for the perceived conservation status of the population. Our results suggest that such assumptions and interpretations may not be valid. Additionally, mitochondrial data are frequently used in more complex ways to estimate contemporary and/or historical population sizes, and fluctuations therein, in attempts to understand the relationships between contemporary and historical abundance and the underlying driving factors (e.g., Roman & Palumbi, 2003). Although such analyses are based on more characteristics of mitochondrial DNA than just diversity, our results still raise questions about the validity of such inferences in cetaceans because if abundance is not closely correlated with patterns of diversity, then it is also not likely correlated with other characteristics of mitochondrial sequences. Of course, mitochondrial characteristics will be informative of mitochondrial effective population size (*N*_*e*_), but it is clear that the relationships between mitochondrial *N*_*e*_ and *N* are complex and that it may be erroneous to assume a clear relationship between the two, and therefore to make direct inferences of *N* based on estimates of *N*_*e*_.

Contrary to the mitochondrial data, population size was positively correlated with diversity at microsatellite loci, with correlation coefficients of 0.425 and 0.703 for the Regional and Rangewide datasets, respectively. These values are comparable to, but somewhat lower than, those found in other taxa. For example, Knaepkens, Bervoets, Verheyen, and Eens (2004) found correlation coefficients of ~0.76 between microsatellite diversity and a proxy of population size in European bullhead (*Cottus gobio*), and Hensen and Oberprieler (2005) obtained a correlation coefficient of 0.8 between genetic diversity at RAPD loci (randomly amplified polymorphic DNA) and population size in the flowering plant species *Dictamnus albus*. This positive correlation between relative nuclear diversity and population size in cetaceans indicates that nuclear loci, rather than mitochondrial loci, may be useful for making inferences of population size, with the caveat that such inferences may be less reliable for cetaceans than within some other taxonomic groups.

Consistent with these patterns, we also found stronger correlations between relative nuclear diversity than mitochondrial diversity with several other metrics related to population size, including *Current IUCN status*,* IUCN status from 1990s*, and *Population trend*. We consider these metrics to be related since population size is used as a criterion in assigning the status of a species by the IUCN (ver 3.1) and, therefore, species with larger populations are less likely to be considered endangered or declining than species with smaller ones. The positive correlation between relative nuclear diversity and *Population trend* was significant for both Regional and Rangewide datasets, lending further support for a positive relationship between relative nuclear diversity and population size. The relatively low correlation between relative nuclear diversity and the two metrics of IUCN status may be due to a small sample size: 53% of cetacean species are listed as “Data Deficient.”

### 4.2 Social structure

We found *Social structure* to be the most important factor in determining mitochondrial genetic diversity in cetaceans (relative to the other factors tested). The importance of social structure in both the Rangewide and Regional mitochondrial genetic diversity models was driven by significantly lower levels in the five cetacean species with known or presumed matrilineal social systems, in the sense that females typically stay grouped with their mothers while both are alive (killer whale—*O. orca*, sperm whale*—P. macrocephalus,* long‐ and short‐finned pilot whale—*Globicephala melas* and *G. macrorhynchus*, and false killer whale—*Pseudorca crassidens*). This pattern has also been found in several other studies (e.g., Alexander et al., 2013; Hoelzel et al., 2002; Whitehead, 1998; Whitehead et al., 2017). Several hypotheses have been proposed regarding mechanisms that could lead to such markedly reduced mitochondrial diversity in these matrilineal species without having a noticeable impact on nuclear diversity. These include historical bottlenecks, selective sweeps within the mitochondrial genome, and cultural hitchhiking. Whitehead et al. (2017) recently considered the likelihood of these data under each hypothesis and concluded that cultural hitchhiking is the most parsimonious. Briefly, bottlenecks seem unlikely because a bottleneck should also reduce relative nuclear diversity, though perhaps not to the same extent. This prediction does not fit well with our dataset because the five matrilineal species do not stand out as outliers with regard to relative nuclear diversity as they do for mitochondrial diversity. While selective sweeps of the mitochondrial genome could result in these patterns, there is not a clear hypothesis regarding why the matrilineal species, specifically, would show such a pattern. The cultural hitchhiking hypothesis, however, predicts this pattern of low mitochondrial diversity within the matrilineal species, with no such reduction in nuclear diversity, as long as mating sometimes occurs between cultural groups. As the scientific community gathers more data, it will become easier to distinguish between the different possible causes of low mitochondrial diversity in the matrilineal cetaceans. Possibly our confidence in the occurrence of cultural hitchhiking will increase or other hypotheses will surface. Nevertheless, as of now and according to our analysis, cultural hitchhiking seems to be the most likely explanation for such differences in the mitochondrial and nuclear genetic diversity of matrilineal species. Other types of social systems present in different cetacean species may also influence patterns of genetic diversity, but perhaps to a lesser degree than matrilineality. However, such patterns have yet to be detected.

### 4.3 Latitudinal range

We found a positive correlation between *Latitudinal range* and mitochondrial genetic diversity. This has been documented in the past for *Drosophila* and fishes (DeWoody & Avise, 2000; Leffler et al., 2012; Ward, Woodwark, & Skibinski, 1994). In the case of cetaceans, this result is not due to a possible positive correlation between *Latitudinal range* and *Population size*: the two factors having low correlation (*r* = .192) and *Population size* not showing a significant relationship with mitochondrial genetic diversity when tested on its own. Instead, this correlation likely results from the segregation of species with large ranges into discrete populations sharing little or no gene flow. Species with wider latitudinal distributions are likely to face a broader range of environmental conditions and contain genetically distinct subpopulations, because of varying selection pressures and genetic drift, leading to higher diversity than species with smaller latitudinal ranges (Ralph & Coop, 2010). Under this scenario, genetic diversity at the species level would increase with latitudinal range since it would incorporate the genetic diversity of all these isolated populations, as well as differences between them.

Given this hypothesis, it is interesting that such a correlation is not also found with relative nuclear diversity. Our interpretation is that this is largely due to the maternal philopatry of most cetaceans. Many cetacean species that are subdivided into multiple populations show markedly stronger differentiation at mitochondrial markers than at nuclear markers, suggesting that population structure often originates from maternally based site fidelity, with males serving as larger conduits of nuclear gene flow between relatively segregated matrilines (Brown Gladden, Ferguson, Friesen, & Clayton, 1999; Hamner, Pichler, Heimeier, Constantine, & Baker, 2012; Hoelzel, 1998). Perhaps the most extreme example of this is the sperm whale, which shows strong mitochondrial differentiation between different areas of the world, but no such differentiation of nuclear markers. This result appears to be due to females showing site fidelity to particular areas, while males may mate in a very different location from their birth (Lyrholm, Leimar, Johanneson, & Gyllensten, 1999). Even within interbreeding populations, and particularly with the baleen whales, there is often seasonal or temporary segregation based on maternal site fidelity, where individuals of different maternal ancestry utilize different feeding areas, resulting in seasonal differentiation of mitochondrial sequences with no such differentiation of the nuclear genome (Baker et al., 1990; D'Intino, Darling, Urbán, & Frasier, 2013).

### 4.4 Habitat

Our analyses found sequentially higher levels of microsatellite diversity in habitat categories “Neritic,” “Oceanic,” and “Both” (Figure 6). Similar results have been found in some fish taxa, with marine fish having higher diversity than their anadromous and freshwater counterparts (Bazin et al., 2006; DeWoody & Avise, 2000; McCusker & Bentzen, 2010; Ward et al., 1994). Such a pattern has not previously been reported for comparisons across cetacean species, although similar patterns have been found within some species. For example, both Hoelzel, Potter, and Best (1998) and Natoli et al. (2004) reported that bottlenose dolphins (*Tursiops truncatus*) belonging to the coastal ecotype had lower levels of genetic diversity than those belonging to the offshore ecotype. Their data suggest that this is likely due to the offshore populations acting as the source of founding individuals for the coastal populations.

Several other hypotheses, which are not mutually exclusive, may also explain this result. We think that the most likely explanation is that oceanic species have had larger historical population sizes due to more continuous, less fragmented environments and more stable historical conditions (DeWoody & Avise, 2000). This would translate into a larger gene pool and, thus, more genetic diversity in oceanic species. Supporting this hypothesis, as well as the patterns found within species, is that coastal environments change much more frequently than oceanic ones, with contemporary coastal habitats being relatively new. The location, size, and conditions of coastal habitats change dramatically with each ice age, which occur relatively frequently (e.g., Calder, 1983). Therefore, contemporary coastal (neritic) populations should represent relatively new founding events from larger, presumably more stable, oceanic ones.

As another hypothesis, Miraldo et al. (2016) suggest that, for terrestrial mammals, species inhabiting regions with more anthropogenic stressors are less genetically diverse. A similar pattern could occur in cetaceans, with the neritic habitat being closer to human populations and under more anthropogenic stress. However, although anthropogenic factors are clearly influencing the abundance and conservation status of *most* cetacean species (e.g., Reynolds et al. 2005), and perhaps levels of genetic variability for some (e.g., Hector's dolphins, *Cephalorhynchus hectori*, Pichler & Baker, 2000), we think that this is an unlikely explanation for this large‐scale pattern. Given the long lifespans and generation times of cetaceans, and the relatively recent developments of large‐scale human exploitation and habitat degradation, it seems unlikely that such recent events could be shaping large‐scale patterns of genetic diversity across cetacean species (e.g., Amos, 1996). Rather, these effects will likely influence genetic characteristics well into the future, as the affected populations slowly move toward new mutation–drift equilibria. This interpretation is supported by the fact that we did not find a strong relationship between genetic diversity and whether or not a species was the subject of intensive whaling (Table 3).

Lastly, social structure could be one of the main drivers of genetic diversity differences between neritic and oceanic species. For example, oceanic dolphins tend to form substantially larger groups than coastal ones and, hence, have access to a larger gene pool. Differences in the levels of philopatry or dispersal patterns between neritic and oceanic species could also account for differences in nuclear genetic diversity (Hoelzel, 1994).

### 4.5 Ocean

Species occurring in both the Atlantic and Pacific Oceans tend to have genetically separated populations inhabiting each ocean. This has been documented for two of the most widely distributed Odontocete species, the bottlenose dolphin (Dowling & Brown, 1993) and the killer whale (Morin et al., 2015) as well as for Mysticetes such as the humpback whales (Jackson et al., 2014). Thus, there will tend to be higher genetic diversity in the “Both” category. While this pattern was expected, the significantly higher relative nuclear genetic diversity in Atlantic species compared to those in the Pacific was unexpected. Possible mechanisms for reduced genetic diversity in the Pacific include lower historical population sizes or sequential founder events in the Atlantic separated by diversification. This result needs to be further investigated by separating species found in the category “Both” into their respective Atlantic and Pacific populations and assessing their historical trends in abundance, distribution, and connectivity (e.g., Alter et al., 2015).

### 4.6 Encephalization quotient

In contrast to *population size*,* encephalization quotient (EQ)* showed a stronger correlation with mitochondrial diversity than relative nuclear diversity. EQ describes the relationship between brain size and body size and is often used as a measure of cognitive ability (Marino, 1998, 2008; Marino, Mcshea, & Uhen, 2004). Previous studies have found that many toothed whales (Odonotocetes) have higher EQ values than expected given general patterns in mammals, with five species having higher EQ values than all primates except humans (Marino, 1998). EQ values are part of the growing body of data hinting at high levels of intelligence in many of the toothed whales.

It is not clear why there seems to be a relationship between Odontocete mitochondrial diversity and *EQ*, and potential links between cognition and genetic diversity have not been much discussed in previous literature. A possible explanation could be that of Wilson (1985) who suggested that large brains allow for innovation and imitation, and, in turn, create an internal pressure to evolve. This would result in an increased genetic diversity through the ability of big brain species to exploit and adapt to new environments or niches, which then subject the population to new evolutionary pressures and change their genetic makeup through the fixation of new mutations, or add to population structure increasing genetic diversity. There is evidence that bigger brained bird and terrestrial mammal species are more successful at colonizing new habitats (Sol, Bacher, Reader, & Lefebvre, 2008; Sol, Timmermans, & Lefebvre, 2002; Sol et al., 2005) and that increased social learning and rate of innovation are both correlated with brain size in birds and primates (Lefebvre, 2013). Although none of these studies mentioned cetaceans, most Odontocete species live in large or stable groups and there is an increasing body of evidence regarding their culture and social learning abilities (Marino et al., 2007; Whitehead and Rendell 2015), which would allow them to share newly acquired ways to exploit a niche via both vertical and horizontal learning and, thus, increase their genetic diversity. This correlation, however, warrants further investigation as EQ values have not yet been calculated for most cetacean species.

## 5 CONCLUSION

There are major differences between the factors influencing mitochondrial and nuclear genetic diversity in cetaceans. While relative nuclear genetic diversity relates strongly to habitat type and seems to follow standard population genetic theory with respect to population size, mtDNA is mostly influenced by social structure and species range. These results indicate that nuclear data should be preferred over mtDNA for making inferences on population size. Moreover, we found that several factors seem to be influencing patterns of diversity in cetaceans, and therefore the interpretation of such data is more complex than is typically appreciated.

## CONFLICT OF INTEREST

None declared.

## AUTHOR CONTRIBUTIONS

FV conducted most of the literature review and data collation, conducted the analyses, and helped writing the manuscript. HW helped with the literature review, data collation, and analyses and also helped write the manuscript. TRF provided guidance with the data analysis and interpretation and helped write the manuscript.
