# Supplementary material for: What factors shape genetic diversity in cetaceans?
Source: Ecol Evol. 2018 Jan 3;8(3):1554–72. doi: 10.1002/ece3.3727 (PMC5792597; doi:10.1002/ece3.3727)
Supplement: Supplementary file 2 [file ECE3-8-1554-s002.docx]

# Appendix S1

**Vachon et al. What factors shape genetic diversity in cetaceans?**

## **Factors potentially predicting genetic diversity: additional procedural information (see Table 1)**

Six demographic factors were included: Logged approximate *Population size* (1), *current IUCN status* (ver 3.1) (2), *IUCN status from the 1990s* (3), *Population trend* (4), *Whaling1* (5) and *Whaling2* (6). *Whaling1* is an index based on the historical whaling information presented in Perry et al. (1999). 0 corresponds to no interactions with the whaling industry, 1: not extensively whaled and 2: heavily exploited. *Whaling2* is a binary variable regarding whether the species has been harvested through whaling or not.

There were two factors based on classification. We compared genetic diversity between Mysticetes and Odontocetes (7), as well as between the different cetacean families (8). Species were classified according to the Society for Marine Mammology’s (Committee on Taxonomy 2016) list of marine mammal species and subspecies.

Six factors related to distribution were also included: *Latitudinal range* (9), *Hemisphere* (10), *Ocean1* (11), *Ocean2* (12), *Habitat* (13) and *Temperature* (14). *Latitudinal range* corresponds to the number of degrees of latitude making up a species’ range according to the maps in Folkens et al. (2002) while *Habitat* corresponds to the IUCN Red List (2016) habitat classification scheme. A score of 9.1-9.10 correspond to neritic habitats and a score of 10.1-10.4 oceanic habitats. We only considered whether the species occurred in neritic and/or oceanic habitat. *Ocean1* is the number of oceans included in the species’ range (up to 5), while *Ocean2* is whether the species is found exclusively in the Atlantic, Pacific or in both. The Indian Ocean was not included in this later factor since none of our cetacean species had a distribution restricted to the Indian Ocean while some species are only found in the Atlantic or Pacific.

The four biological factors included were: *Maximum female length* (15), *Generation time* (16), *Lifespan* (17) and *Encephalization Quotient* (EQ_0.67_) (18). EQ gives residuals from a regression of log brain weight on log body weight (Jerison 1973)_._ EQ_0.67_ is the most commonly used EQ measure and corresponds to how much larger or smaller than expected the animals’ brain is compared to the regression line. Hence, an EQ_0.67_ value above 1 means that the brain is bigger than would be expected according to body size (Jerison 1973). We restricted this factor to Odontocetes since Mysticetes have disproportionate body enlargement, making EQ an inappropriate measure (Marino 2008). We refer to EQ_0.67_ as EQ.

We considered three behavioural factors: *Group size* (19), *Mysticeti breeding strategy* (20) and *Social structure* (21). The social structure factor differentiates matrilineal and non-matrilineal species. A species was considered matrilineal if the female offspring typically remain grouped with their mothers for their entire lifetime (Whitehead 1998). We recognized five cetacean species as matrilineal: *Orcinus orca* (killer whale), *Physeter macrocephalus* (sperm whale), *Globicephala macrorhynchus* (short-finned pilot whale), *Globicephala melas* (long-finned pilot whale) and *Pseudorca crassidens* (false killer whale) (Whitehead et al. 2017).

In many cases, sample size of species was reduced because some of the information was unavailable for some species for which we have genetic diversity data.

**Table S1 (TableS1.htm). Cetacean species with latitude ranges, approximate population sizes and estimates of mitochondrial D-loop nucleotide diversity (π), and nuclear genetic diversities (mic) using microsatellites (with estimated standard errors), for both rangewide (O) and regional (R) estimates. This Table is an expansion of the table in Whitehead et al. (2017), to include the value of each factor for each species.**

**REFERENCES**

Committee on Taxonomy (2016) List of marine mammal species and subspecies. Society for Marine Mammalogy. http://www.marinemammalscience.org. Accessed September 2016.

IUCN (2016) 2016 IUCN red list of threatened species. http://www.iucnredlist.org. Accessed September 2016.

Jerison, H. J. (1973). Evolution of the brain and intelligence. New York: Academic Press.

Marino, L. (2008). Encyclopedia of marine mammals (2nd ed). Amsterdam: Elsevier Academic Press. Chapter 37, Brain size evolution; p.149-152.

Perry, S. L., Demaster, D. P., Silber, G. K. (1999). The great whales: history and status of six species listed as endangered under the US Endangered Species Act of 1973. Marine Fisheries Review, 61, 1–74.

Folkens, P., Reeves, R. R., Stewart, B., Clapham, P., & Powell, J. (2002) Guide to marine mammals of the world (2nd ed.). New York: A.A. Knopf.

Whitehead, H. (1998). Cultural selection and genetic diversity in matrilineal whales. Science, 282, 1708-1711.

Whitehead, H., Vachon, F., & Frasier, T. (2017). Cultural hitchhiking in the matrilineal whales. Behavior Genetics, 47, 324-334. doi:10.1007/s10519-017-9840-8
